# Current Concepts in Pediatric Obstructive Sleep Apnea

**DOI:** 10.3390/children10030480

**Published:** 2023-03-01

**Authors:** Manisha Witmans, Mary Anne Tablizo

**Affiliations:** 1 Department of Pediatrics, Faculty of Medicine & Dentistry, University of Alberta, Edmonton, AB T6G 2B7, Canada; 2 Department of Pediatrics, Stanford University School of Medicine, Palo Alto, CA 94304, USA; 3 Department of Pediatrics, Valley Children’s Hospital, Madera, CA 93636, USA

Obstructive sleep apnea (OSA) is described as intermittent partial or complete upper airway obstruction that can disrupt respiratory and ventilatory patterns during sleep [1]. In the pediatric population, OSA has an estimated prevalence of 1 to 4%. OSA has been shown to have a negative impact on the behavior, cognitive performance, emotional regulation and growth of children. The long-term sequelae from untreated OSA are associated with cardiovascular, endothelial and metabolic disorders [2]. This special issue specifically focuses on the various aspects related to the clinical presentation, objective findings, risk factors, associated comorbid conditions, sequelae, pathophysiology, diagnosis and management of OSA in children from the neonates to the adolescent age group. Dr. Ron Harper and colleagues discuss the implications of intermittent hypoxia on insulin secretion in rodents and its implications for pediatric OSA [3]. Clinically, the association of OSA with asthma and hepatic steatosis in children and adolescents is reported here [4,5]. OSA in high-risk populations such as Down syndrome, neonates, and children with attention deficit hyperactivity disorder are also addressed in this special edition [6,7,8,9]. 

The predisposing factors and clinical presentation for OSA are different in each age group. The airway can be compromised structurally or functionally or both which can affect anyone from birth until adulthood. Neonates have distinctive anatomic and physiologic features that predispose them to have OSA compared to older children and adults. OSA is most commonly caused by airway abnormalities extending from the nose to the larynx mainly related to craniofacial malformations, neuromuscular disorders, and prematurity in neonates [7]. In young healthy children, the main etiology of OSA is related to adenoid and/or tonsillar hypertrophy [10]. However, other factors such as malocclusion or other causes of inflammation such as allergic rhinitis and asthma may also play a significant role [10,11]. Obesity is increasingly more prevalent in the pediatric population and is a significant risk factor for OSA especially in the adolescent group [10]. Comprehensive sleep history and thorough physical examination to screen for OSA in children are inadequate for a definitive diagnosis of OSA. Despite the extensive clinical description of symptoms and signs of OSA, the sensitivity and specificity of these parameters are limited in predicting polysomnography-confirmed OSA [12]. Attempts to develop different screening tools have resulted in limited sensitivity and specificity. In-laboratory polysomnography (PSG) remains the gold standard for diagnosing pediatric sleep-disordered breathing [12]. There are however limitations related to the attended in-lab PSG, such as limited access to a sleep center, the specialized training involved in studying children, the laborious nature of the test, and social/economic barriers, which can delay diagnosis and treatment. There have been increasing studies on the validation and utilization of alternative methods of diagnosis of OSA in children including home sleep testing and wearable devices [12]. Due to the lack of sufficient evidence, AASM’s most recent position statement still recommends PSG for diagnosis of OSAin children [12]. However, as the tools continue to become more sophisticated, particularly wearables, some of these challenges should improve. 

Given the sequelae associated with untreated OSA, this series highlights the importance of timely and effective treatment of OSA. Adenotonsillectomy (AT) is the first-line treatment in children with enlarged tonsils and adenoids [13]. The predictors for post AT respiratory complications requiring intervention are children with severe OSA particularly AHI > 40/h, younger children (<2 years of age), lower oxygen saturation (SpO_2_), and poor nutritional status which was shown in a retrospective review conducted by Saeid et al. [14]. For children where surgery is not indicated or desired, or for those with postoperative residual OSAS, positive airway pressure (PAP) therapy can be an effective treatment [15]. Benke et al. study showed that the use of autoCPAP is effective and safe for the treatment of OSAS in pediatric patients with obesity [16]. Using autoCPAP in children with obesity can avoid delay in treatment; however additional research is needed to verify the long-term effectiveness of autoCPAP in children with OSA. Adjuvant nonsurgical therapy is available for the treatment of OSA in children such as medical management with the use of anti-inflammatory medications, positional therapy, myofunctional therapy, and evolving use of dental devices such as rapid maxillary expansion and oral devices. There are also adjuvant surgical procedures such as lingual tonsillectomy and recently hypoglossal nerve stimulation device has been used to treat older children with Down syndrome with refractory OSA post-AT and unable to tolerate PAP therapy [6]. The literature on these additional treatment options is still limited.

In the pivotal Childhood Adenotonsillectomy Trial (CHAT study), children 5–9 years with OSA without prolonged oxyhemoglobin desaturations (mild obstructive sleep apnea) were randomly assigned to adenotonsillectomy (AT) or watchful waiting with supportive care (WWSC) [17]. The primary outcomes were to document changes in attention and executive function evaluated with the Developmental Neuropsychological Assessment (NEPSY). Although adenotonsillectomy helped improve behavior, quality of life and PSG findings, there were some children who also did well in the WWSC group which suggests that OSA can spontaneously resolve. Magnusdottir and colleagues performed secondary analyses of the results from the CHAT study to determine if there are factors that could predict which children with mild OSA are likely to have spontaneous resolution of OSA using cardiopulmonary coupling signals based on symptoms and PSG. They showed that those with higher baseline sleep quality, mild OSA, normal body weight, higher executive function score, and better quality of life were more likely to have spontaneous resolution of OSA [14]. Clearly, this needs to be evaluated prospectively. Although AT is the first line of therapy, the CHAT study and associated data bring into question who actually needs and benefits from surgery. This has become a significant concern because surgery may result in a cure in some children and is less likely to be curative in children with obesity or comorbid asthma. If the cardiopulmonary coupling can be assessed apriori, this could result in a fundamental shift in the way childhood OSA is managed and treated as the authors point out. Dr. Guillemineault and colleagues had shown that children with OSA required both rapid maxillary expansion and adenotonsillectomy for a complete cure of OSA in children with malocclusion and associated OSA symptoms [11]. Preventative care to optimize the normal development of maxillofacial skeleton and function to decrease risk of developing OSA is also discussed. Advances in oral appliances for children continue to evolve which indicates the importance of interdisciplinary synergistic collaboration with the field of dentistry and medicine to treat OSA [11]. There is a need for increased awareness and education about pediatric OSA in both medical and dental schools [18].

The journey of learning about pediatric OSA is ongoing and we continue to take steps forward. Management of inflammation in the airway is an important part of the treatment of OSA. Evolving technology that is accessible, portable, easy to use, robust, and with great validity is being developed to not only screen but diagnose OSA with ease and possibly other sleep disorders. As the changes in sleep medicine from artificial intelligence, machine learning, wearable technology for diagnosis, even possibly robotic surgery, and accessible pathways for diagnosis and management continue to accelerate, the field of sleep medicine will see dramatic changes. The diagnosis and management of pediatric OSA are expected to evolve as we continue to find newer approaches to address the problem using personalized medicine.

We thank the tireless efforts of the contributors to make this series of articles enjoyable for your journey in learning about pediatric OSA and the team at Children for giving us the opportunity to share this work with you.

## References

[1] American Academy of Sleep Medicine (2014). International Classification of Sleep Disorders.

[2] Thomas S., Patel S., Gummalla P., Tablizo M.A., Kier C. (2022). You Cannot Hit Snooze on OSA: Sequelae of Pediatric Obstructive Sleep Apnea. Children.

[3] Pae E.-K., Chung M.-K., Harper R.M. (2022). Intermittent Hypoxia Interferes with Autocrine Effects of GABA on Insulin Secretion in Postnatal Rodents—Implications for Pediatric Obstructive Sleep Apnea. Children.

[4] Garza N., Witmans M., Salud M., Lagera P.G.D., Co V.A., Tablizo M.A. (2022). The Association between Asthma and OSA in Children. Children.

[5] Carotenuto M., Di Sessa A., Esposito M., Grandone A., Marzuillo P., Bitetti I., Umano G.R., Precenzano F., del Giudice E.M., Santoro N. (2021). Association between Hepatic Steatosis and Obstructive Sleep Apnea in Children and Adolescents with Obesity. Children.

[6] Gastelum E., Cummins M., Singh A., Montoya M., Urbano G.L., Tablizo M.A. (2021). Treatment Considerations for Obstructive Sleep Apnea in Pediatric Down Syndrome. Children.

[7] Chandrasekar I., Tablizo M.A., Witmans M., Cruz J.M., Cummins M., Estrellado-Cruz W. (2022). Obstructive Sleep Apnea in Neonates. Children.

[8] Urbano G.L., Tablizo B.J., Moufarrej Y., Tablizo M.A., Chen M.L., Witmans M. (2021). The Link between Pediatric Obstructive Sleep Apnea (OSA) and Attention Deficit Hyperactivity Disorder (ADHD). Children.

[9] Di Mauro P., Cocuzza S., Maniaci A., Ferlito S., Rasà D., Anzivino R., Vicini C., Iannella G., La Mantia I. (2021). The Effect of Adenotonsillectomy on Children’s Behavior and Cognitive Performance with Obstructive Sleep Apnea Syndrome: State of the Art. Children.

[10] Saint-Fleur A.L., Christophides A., Gummalla P., Kier C. (2021). Much Ado about Sleep: Current Concepts on Mechanisms and Predisposition to Pediatric Obstructive Sleep Apnea. Children.

[11] Heit T., Tablizo B.J., Salud M., Mo F., Kang M., Tablizo M.A., Witmans M. (2022). Craniofacial Sleep Medicine: The Important Role of Dental Providers in Detecting and Treating Sleep Disordered Breathing in Children. Children.

[12] Kang M., Mo F., Witmans M., Santiago V., Tablizo M.A. (2022). Trends in Diagnosing Obstructive Sleep Apnea in Pediatrics. Children.

[13] Uwiera T.C. (2021). Considerations in Surgical Management of Pediatric Obstructive Sleep Apnea: Tonsillectomy and Beyond. Children.

[14] Saied N., Solis R.N., Funamura J., Chen J., Lammers C., Nandalike K. (2022). Clinical Characteristics and Post-Operative Outcomes in Children with Very Severe Obstructive Sleep Apnea. Children.

[15] Hady K.K., Okorie C.U.A. (2021). Positive Airway Pressure Therapy for Pediatric Obstructive Sleep Apnea. Children.

[16] Benke S., Okorie C.U.A., Tablizo M.A. (2021). The Use of Auto-Titrating Continuous Positive Airway Pressure (AutoCPAP) for Obstructive Sleep Apnea Syndrome in Children with Obesity. Children.

[17] Magnusdottir S., Hilmisson H., Raymann R.J.E.M., Witmans M. (2021). Characteristics of Children Likely to Have Spontaneous Resolution of Obstructive Sleep Apnea: Results from the Childhood Adenotonsillectomy Trial (CHAT). Children.

[18] Alharbi L.N., Alsaikhan M.A., Ali S.N.A.-H., Farah R.I. (2021). Pediatric Obstructive Sleep Apnea: Knowledge and Attitudes of Medical and Dental Students and Fresh Graduates from Saudi Arabia. Children.

